# Genetic Diversity, Demographic Parameters, and Trophic Ecology of the Pampas Cat (*Leopardus garleppi*) in a Ramsar Wetland of Northwestern Peru

**DOI:** 10.3390/genes17030320

**Published:** 2026-03-16

**Authors:** Manuel Santiago-Plata, Jennifer Adams, Janet L. Rachlow, Cindy M. Hurtado, Alvaro Garcia-Olaechea, Taal Levi, Lisette P. Waits

**Affiliations:** 1 Department of Fish and Wildlife Sciences, University of Idaho, Moscow, ID 83844, USA; vsantiagoplata@uidaho.edu (M.S.-P.); adamsj@uidaho.edu (J.A.); jrachlow@uidaho.edu (J.L.R.); 2 Department of Forest Resources Management, University of British Columbia, Vancouver, BC V6T 1Z4, Canada; cindymeliza@gmail.com; 3 Centro de Investigación Biodiversidad Sostenible—BioS, Piura 20001, Peru; agarolae@yahoo.com; 4 Department of Fisheries, Wildlife, and Conservation Sciences, Oregon State University, Corvallis, OR 97331, USA; taal.levi@oregonstate.edu

**Keywords:** population genetics, population size, effective population size, microsatellite loci, genetic bottleneck, kinship analysis, noninvasive genotyping, DNA metabarcoding, conservation genetics, *Leopardus garleppi*

## Abstract

**Background/Objectives**: Habitat degradation and fragmentation reduce population size, genetic diversity, and connectivity, increasing extinction risk in small and isolated populations. Coastal wetlands of northwestern Peru have undergone extensive anthropogenic modification, yet the genetic and ecological status of resident carnivore populations remains poorly documented. This study aimed to assess genetic diversity, relatedness, demographic signals, and diet composition of a Pampas cat (*Leopardus garleppi*) population inhabiting the Mangroves San Pedro de Vice (MSPV), a Ramsar-listed coastal wetland. **Methods**: We combined noninvasive fecal genotyping using eight nuclear microsatellite loci with vertebrate DNA metabarcoding. Scat samples were collected across three field seasons (2019–2021). Individual identification, genetic diversity metrics, genetic mark–recapture estimation of census size (Nc), effective population size (Ne), bottleneck tests, and relatedness analyses were performed to evaluate population status and kin structure. Dietary composition was characterized using metabarcoding and assessed for sex-specific differences. **Results**: Sixty-eight scats yielded multilocus genotypes for nine individuals (six males, three females). Genetic analyses revealed moderate diversity (mean allelic richness = 3.47; observed heterozygosity = 0.69; expected heterozygosity = 0.58) and evidence consistent with a recent genetic bottleneck. Genetic mark–recapture analyses estimated a small census size (Nc = 9; 95% CI: 7.0–9.0), while the effective population size was markedly low (Ne = 2.4; 95% CI: 1.5–7.4), yielding an Ne/Nc ratio of ~0.27. Multiple first-order kin dyads were detected, indicating strong local kin structure and limited external recruitment. Metabarcoding identified eight vertebrate prey species, with diet dominated by the native rodent *Aegialomys xanthaeolus*. No significant sex-specific differences in diet composition were detected. **Conclusions**: The MSPV Pampas cat population represents a small, kin-structured range-edge population showing signatures consistent with recent genetic erosion and restricted connectivity. These patterns align with isolation in a degraded coastal wetland landscape, highlighting the importance of habitat protection, prey resource conservation, and restoration of functional connectivity to support long-term population persistence.

## 1. Introduction

Genetic diversity constitutes a fundamental component of biodiversity, serving as a critical buffer against environmental perturbations and enabling populations to adapt to changing conditions and new stressors [1,2]. In small or isolated populations, losing allelic richness and heterozygosity lowers evolutionary potential, making them more vulnerable to extinction caused by inbreeding depression and random demographic events [3,4]. Additionally, populations at the edge of their range are often more vulnerable, typically showing less genetic variation due to founder effects and limited gene flow [5,6]. The maintenance of genetic diversity is therefore essential for sustaining adaptive capacity and long-term viability, especially in the context of habitat fragmentation and climate change [7,8]. In this sense, conservation strategies that fail to preserve genetic variation may inadvertently accelerate declines in fitness and population persistence, ultimately undermining recovery efforts for threatened species [9].

Understanding the genetic processes that influence population persistence requires examining gene flow, inbreeding, and genetic bottlenecks, all of which affect the retention or loss of genetic variation across generations [7,10]. In this context, gene flow improves connectivity and counteracts genetic drift, while restricted gene flow promotes inbreeding and the expression of harmful alleles [11,12]. Further, populations with limited dispersal or habitat fragmentation often show higher relatedness and lower heterozygosity, which are signs of recent demographic decline or isolation [5,13]. In addition, genetic bottlenecks, detectable through heterozygosity excess or shifts in allele frequency distributions, offer further insight into demographic instability [14,15]. These signatures are vital for identifying populations at risk of genetic erosion and prioritizing conservation efforts aimed at restoring gene flow and reducing inbreeding depression [9,16].

Reliable estimates of population size, sex ratio, and individual persistence are fundamental to evaluating the status of wildlife populations, particularly elusive carnivores inhabiting fragmented or remote environments [17,18]. Noninvasive genetic monitoring enables such assessments by linking repeated detections of individuals across years to infer abundance, site fidelity, and sex composition [19,20]. These demographic attributes are key for identifying population trends and potential reproductive biases that may compromise viability. For example, deviations from balanced sex ratios or low interannual recaptures may reflect a combination of demographic processes (e.g., reduced apparent survival, temporary emigration, detection heterogeneity, or limited recruitment/immigration), particularly in species with sex-biased movement patterns [21,22,23,24]. Thus, combining demographic information with genetic diversity metrics provides a more comprehensive view of population stability and facilitates the early detection of decline in small, isolated carnivore populations [25].

Beyond diversity estimates, analyses of pairwise genetic relatedness reveal patterns of dispersal limitation, mating structure, and social organization in species traditionally considered solitary [10,26]. Additionally, high relatedness and frequent first-order kin relationships within a small area may suggest high site fidelity and reduced immigration, which are common in fragmented systems [12,27]. Such kin clustering can increase inbreeding and reduce adaptive potential, particularly when populations are isolated by natural or anthropogenic barriers [1,9,28]. In this context, noninvasive genetic methods using fecal DNA provide a valuable way to assess these processes while minimizing disturbance, enabling the simultaneous determination of individual identity, relatedness, and temporal persistence [29,30].

Along with demographic parameters, dietary ecology provides important context for understanding the ecological flexibility and resource dependencies of small carnivores. In this context, DNA metabarcoding of fecal samples offers precise identification of prey taxa and has become essential for assessing the trophic niches of elusive species [31,32]. In fragmented habitats such as coastal wetlands and arid mosaics, diet composition can reveal how local prey communities influence predator foraging behavior and resilience to habitat changes [33,34,35]. Furthermore, combining diet data with genetic monitoring helps clarify how kin-structured populations divide resources and maintain coexistence under ecological constraints [36,37]. This integrated approach enhances conservation efforts by linking genetic and ecological information to evaluate both the evolutionary potential and the functional sustainability of populations at the edges of species.

The Pampas cat (*L. garleppi* [Matschie 1912]) is a small wild felid of ~2–5 kg [38], formerly considered a subspecies of *L. colocola* (Molina 1782) but recently recognized as a distinct species [39]. Its distribution spans both slopes of the Andes, from southern Colombia to northern Argentina and Chile [39,40], encompassing a remarkable diversity of habitats that include high-elevation ecosystems such as páramo and puna, as well as dry forests, cerrado, deserts, wetlands, and agricultural landscapes [41,42]. In Peru, *L. garleppi* occurs along both Andean slopes from sea level to nearly 5000 m.a.s.l. [43,44]. However, coastal records are exceptionally scarce and largely confined to isolated wetlands and arid desert remnants [44,45]. The confirmed presence of *L. garleppi* in the Mangroves San Pedro de Vice (MSPV) on Peru’s northwestern coast [46] is therefore highly significant, representing the only documented population inhabiting a mangrove ecosystem. This atypical lowland occurrence contrasts sharply with the species’ predominantly montane distribution, offering a unique opportunity to explore its ecological plasticity and potential local adaptation within a coastal environment.

Due to the importance of understanding ecological and evolutionary dynamics across the species’ range, particularly between central Andean populations and peripheral groups in marginal habitats, this study assessed demographic parameters, genetic diversity, and dietary composition of the Pampas cat population at MSPV in northwestern Peru using fecal DNA sampling. Our objectives were (a) quantify genetic diversity and compare it with estimates from other populations; (b) estimate census population size (Nc), effective population size (Ne), and annual minimum population size; (c) evaluate genetic evidence of recent bottlenecks, inbreeding, and pairwise relatedness among individuals; (d) estimate apparent sex ratio and interannual recaptures; and (e) characterize dietary composition through DNA metabarcoding analysis.

We hypothesized that the MSPV functions as a closed population with limited immigration, resulting in reduced genetic diversity relative to other *L. garleppi* populations. Accordingly, we expected the population to be small in both census and effective size, with a low effective population size, elevated relatedness, and signals of a recent bottleneck. We further anticipated sex-specific variation in apparent sex ratio and persistence driven by movement ecology, with greater interannual variability in male detections and higher site fidelity in females. Under this scenario of limited immigration and small population size, we predicted relatively high interannual recapture rates, with most individuals identified in 2019–2020 expected to be detected again in 2021. Finally, we hypothesized that diet would be dominated by avian and rodent prey, with males consuming a broader prey spectrum than females, consistent with sex-based differences in space use documented in small felids [47,48,49,50].

We tested these hypotheses using a noninvasive genetic sampling approach based on fecal DNA, a well-established method for studying elusive carnivores that enables the simultaneous collection of genetic and ecological information without direct animal handling [51,52]. Our findings provided essential baseline data on the Pampas cat population inhabiting the MSPV, an ecologically significant coastal wetland at the northwestern limit of the species’ range. The insights gained are critical for informing targeted conservation and management actions, particularly given the site’s limited connectivity, ecological fragility, and the scarce documentation of this species in arid coastal habitats.

## 2. Materials and Methods

### 2.1. Study Area

**Figure 1:**
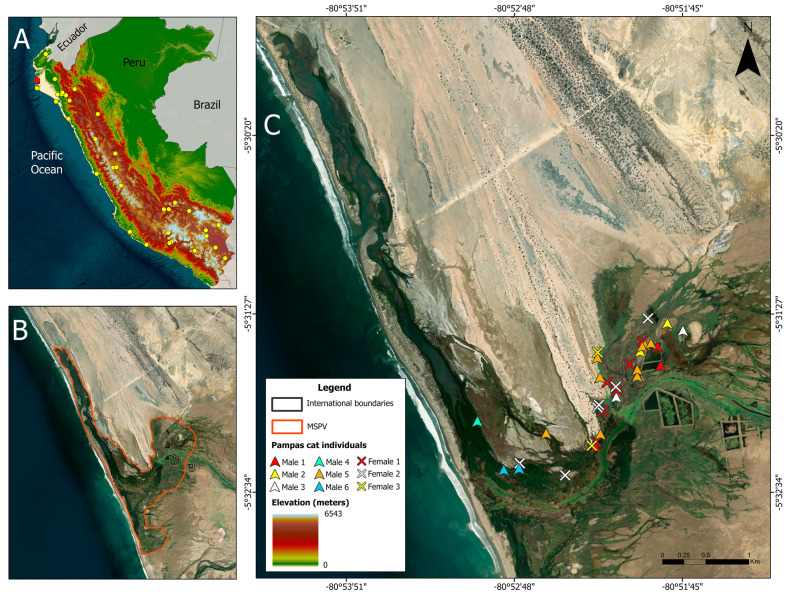
Distribution and study site of the Pampas cat (*L. garleppi*) in Peru. Panel (**A**) shows historical occurrence records (yellow circles; [44]) across the species’ elevational range (0–6543 m), highlighting its association with both Andean and lowland habitats. The red dot marks the location of the Mangroves San Pedro de Vice (MSPV). Panel (**B**) shows the delimitation of the MSPV (orange outline) along the northwestern coast of Peru. Panel (**C**) shows a detailed view of the MSPV, with individual Pampas cats identified through noninvasive genetic sampling (colored symbols). International boundaries are included for geographic context.

The MSPV is situated on the Pacific coast in the district of Vice, Sechura Province, Piura, Peru (Figure 1; 05°30′34″ S, 80°52′53″ W), covering roughly 3013 hectares [46]. This coastal wetland exhibits a patchwork of ecosystems, forming a boundary between freshwater and marine environments [53]. Inland, it is bordered by the Sechura Desert ecoregion, a hyper-arid zone known for low rainfall and minimal vegetation [46]. Recognized as a Wetland of International Importance under the Ramsar Convention, the MSPV is the southernmost remaining mangrove patch along the Pacific coast of South America [54,55]. The area functions as a crucial breeding and refuge area for aquatic species of commercial importance [56], while also hosting diverse migratory bird populations and serving as an important stopover point along transcontinental flyways [57,58]. The average elevation of MSPV is less than five meters above sea level (m.a.s.l.), and its climate is heavily influenced by the nearby Sechura Desert, known for its extreme dryness [59]. Annual rainfall varies from 26 to 50 mm, with temperatures ranging from 23.3 °C to 27.2 °C [46,60]. The mangrove stands are dominated by *Laguncularia racemosa* and *Avicennia germinans*, surrounded by sparse desert vegetation such as *Prosopis pallida*, grasses like grama, and *Ceratonia siliqua* [61]. These ecological features, along with the presence of the uncommon pampas cat population, make MSPV a key site for studying adaptation, isolation, and habitat utilization in edge populations of Andean mammals.

### 2.2. Fecal DNA Collection and Extraction

We employed a noninvasive genetic sampling approach to collect fecal samples of the Pampas cat for genetic analysis. Sampling was conducted across three distinct periods: February–May 2019, February–September 2020, and March–April 2021, during which a total of 142 fecal samples were collected (see Results for annual details). Fecal material was obtained from Pampas cat latrines and cavities in the ground made by feral pigs along the MSPV, based on characteristic visual and olfactory signs such as appearance, color, and odor. We collected a small amount of fecal material (~0.5 mL) and transferred it into a sterile 2 mL screw-cap tube containing 1.4 mL of dimethyl sulfoxide saline solution (DETs buffer: 20% dimethyl sulfoxide, 0.25 MEDTA, 100 mM Tris, pH 7.5, with NaCl added to saturation [62]. Each tube was labeled with a unique sample ID, collection date, and GPS coordinates of the sampling location. This procedure was repeated for every fecal sample collected. All samples were maintained in the DETs buffer at room temperature for four to eight months before DNA extraction.

DNA extractions were conducted in a specialized low-quantity DNA room within the Laboratory for Ecological, Evolutionary, and Conservation Genetics (LEECG) at the University of Idaho (Moscow, ID, USA). This facility is specifically designed for noninvasive genetic research, providing rigorous controls to prevent contamination when handling samples with low DNA concentrations. Extractions were carried out using the QIAamp^®^ DNA Stool Mini Kit (Qiagen Inc., Hilden, Germany), following the manufacturer’s protocol. Negative controls were incorporated into each extraction batch to detect potential contamination [52]. Purified DNA samples were stored at −20 °C for later analysis.

### 2.3. Microsatellite Amplification and Genotyping

We screened 26 microsatellite loci originally developed for the domestic cat (*Felis catus* [63,64]) and validated for neotropical and temperate felids by [65,66,67,68]: F53, F98, F124, F146, FCA008, FCA031, FCA043, FCA045, FCA057, FCA075, FCA082, FCA090, FCA096, FCA098, FCA117, FCA124, FCA126, FCA132, FCA205, FCA225, FCA229, FCA275, FCA294, FCA391, FCA441, FCA741. Additionally, we tested two molecular sex markers—AMEL and Zinc Finger [69]. Microsatellite loci suitability was evaluated by examining allelic diversity and probability of identity for unrelated individuals and siblings [70]. Additionally, Polymerase Chain Reaction (PCR) amplification success, genotyping accuracy, and error rates were assessed to ensure the performance of the selected loci [65].

Following initial screening, 15 microsatellite loci and the AMEL sex marker were excluded from further analyses due to their poor amplification efficiency and inconsistent genotyping results. The remaining 11 nuclear DNA microsatellite loci were combined into a single multiplex PCR (Table S1) and labeled with fluorescent dyes (F124-PET [64], FCA008-FAM, FCA031-VIC, FCA045-VIC, FCA075-VIC, FCA096-FAM, FCA117-FAM, FCA126-PET, FCA132-NED, FCA294-PET, FCA391-NED [63]. Sex identification of pampas cat samples was performed using Zinc Finger primers (forward and reverse) developed for domestic cats [69]. Negative and positive controls were incorporated in each PCR batch to assess contamination and ensure reagent quality. Details on PCR components and thermal cycling conditions are described in the Supplementary Materials.

Amplified PCR products were visualized using an Applied Biosystems 3130xl capillary sequencer (Applied Biosystems Inc., Foster City, CA, USA), with GeneScan 500 LIZ (Applied Biosystems Inc., Foster City, CA, USA). Allele calling was performed using GENEMAPPER v6.0 software (Applied Biosystems, Carlsbad, CA, USA). We implemented a two-step multitube approach [71] to discard low-quality nuclear DNA (nDNA) samples. We initially performed PCR in duplicate for each sample, removing those with <40% amplification across the 11 microsatellite loci. Samples amplifying at ≥40% threshold (>5 loci) were retained, and additional PCR replicates were conducted to establish consensus genotypes, with a maximum of six replicates per sample per locus.

We used a Microsoft Access-based application to assess consensus genotypes by comparing replicate profiles. A heterozygous genotype was confirmed when each allele was observed in at least two PCRs, whereas three replicates were required to confirm homozygous genotypes [67]. Individual identification and discriminatory power of the 11 microsatellite loci were assessed using the methodology outlined by [70]. Cumulative probabilities of identity for unrelated individuals (P_(ID)_) and siblings (P_(ID)sibs_) were calculated using GenAlEx v6.5 [72]. We discarded samples that did not achieve a P_IDsibs_ < 0.01 [73], and six loci were required to distinguish between individuals. Genotyping error rates, including allelic dropout (ADO) and false alleles (FA), were assessed by identifying genotype inconsistencies across replicate PCRs [74]. Only heterozygous loci were used to calculate ADO rates, whereas FA rates were estimated from all consensus genotypes. We used MICRO-CHECKER v2.2.3 [75] with the Brookfield 1 estimator to detect null alleles. The sex ratio of the *L. garleppi* population was determined by calculating the proportion of genetically identified males and females across all sampling years. After confirming individual identities through multilocus genotyping, the number of males and females was counted to describe the overall sex ratio and their distribution over time.

### 2.4. Diet Metabarcoding Sequencing Data

To characterize trophic ecology, we selected 53 fecal samples from the 68 successfully genotyped samples (48% of 142 total collected), representing nine sex-identified Pampas cats in the MSPV for dietary analysis (females: F1 = 10, F2 = 8, F3 = 2; males: M1 = 7, M2 = 2, M3 = 5, M4 = 7, M5 = 10, M6 = 2). The laboratory procedures followed a vertebrate DNA metabarcoding workflow, which was adapted from the methodologies established by [32,76]. We extracted DNA from fecal samples in a dedicated pre-PCR facility using a modified Qiagen DNeasy Blood and Tissue Kit protocol (Qiagen, Hilden, Germany), with one extraction blank per batch to monitor contamination. In a separate facility, each sample was amplified in triplicate targeting a ~100 bp fragment of the mitochondrial 12S rRNA gene using the 12S-V5 forward primer (TTAGATACCCCACTATGC; [77]) and a modified reverse primer (YAGAACAGGCTCCTCTAG; [32]). All primers carried unique dual-matching 8 bp indexes (384 total), which enabled identification of sample replicates post-pooling and mitigated tag-jumping [78]. The PCR (20 µL) contained 10 µL AmpliTaq Gold 360 Master Mix (Thermo Fisher Scientific, Waltham, MA, USA), 3 µL water, 5 µL primer mix (200 nM final concentration), and 2 µL DNA template. Three no-template controls were included per plate to quality control step. Thermocycling involved an initial denaturation at 95 °C for 10 min; followed by 35 cycles of 30 s at 95 °C, 30 s at 58 °C, 1 min at 72 °C; and a final extension of 7 min at 72 °C.

Amplicon concentrations were quantified using the AccuBlue High Sensitivity dsDNA Quantitation Kit (Biotium, Fremont, CA, USA) and a fluorescence plate reader. Replicates were normalized to the plate-average DNA concentration to ensure even sequencing depth, and 5 µL per PCR replicate was pooled using an Opentrons OT-2 liquid handler (Opentrons, Brooklyn, NY, USA). Pools from four plates were combined into libraries prepared with the NEBNext Ultra II DNA Library Prep Kit (New England Biolabs, Ipswich, MA, USA). Library concentrations were measured using the AccuGreen Broad Range dsDNA Quantitation Kit and a Qubit Fluorometer (Thermo Fisher Scientific, Waltham, MA, USA). Final pools were quantified with the KAPA Library Quantification Kit (Roche, Basel, Switzerland) at the Center for Quantitative Life Sciences (Oregon State University), equimolarly combined, and sequenced on an Illumina NextSeq 2000 platform (Illumina, San Diego, CA, USA) using 2 × 150 bp PE.

Demultiplexed reads were clustered at 100% similarity, and taxonomic assignment was conducted using LocaTT [79] against the MIDORI2 reference database [80]. Best matches were retained based on BLAST searches performed using the NCBI BLAST platform (https://blast.ncbi.nlm.nih.gov/Blast.cgi, accessed on 12 May 2025). A potential prey species list for the Pampas cat was then applied to prioritize plausible local taxa. Assignments with ≥99% query coverage and 100% sequence identity were resolved to the species level. Finally, we cross-referenced the resulting prey taxonomic list with published records of bird and small mammal species [57,81] documented in the MSPV. For subsequent analyses, sequence filtering and quality control followed a multi-step approach: (i) removing sequences representing ≤ 0.5% of the total reads within a replicate to eliminate PCR or sequencing errors and minimize potential contamination from tag-jumping or ambient DNA, and (ii) excluding taxa detected in only one of the three PCR replicates.

### 2.5. Population Genetic Metrics and Comparative Diversity Framework

Departures from Hardy–Weinberg equilibrium (HWE) were evaluated by estimating the Weir and Cockerham’s inbreeding coefficient (F_IS_) among microsatellite loci. In addition, linkage disequilibrium (LDE) was assessed between all pairs of loci using the R package genepop v1.2.2 [82]. To correct for multiple testing, false discovery rate (FDR) adjustments were applied using the Benjamini–Hochberg procedure [83]. Additionally, we estimated standard measures of genetic diversity, including the number of alleles (Na), observed heterozygosity (Ho), and expected heterozygosity (He) per locus and across loci using GenAlEx [72]. Allelic richness (AR) was estimated via rarefaction method using HP-RARE v1.0 [84].

To compare mean He in the MSPV population with estimates from other regions, we compiled a per-locus dataset including the four microsatellite loci shared across studies (FCA031, FCA045, FCA096, and FCA294; [85]). Sampling localities of Pampas cats belong to four main regions, including the MSPV: central Peru (Ancash, *n* = 19; Yauyos/Canchayllo, *n* = 19; Junin National Reserve, *n* = 12; Tacna/Puno, *n* = 19), northwestern Peru (MSPV, *n* = 9), western Bolivia (La Paz/Oruro, *n* = 12; Potosí, *n* = 15), and northern Argentina (Jujuy, *n* = 28; Catamarca/Salta, *n* = 30). Only sites with sample sizes ≥ 9 individuals were retained to ensure comparability among populations. Because sample sizes varied among locations and the assumption of normality was not met, we applied a nonparametric Friedman test, treating loci as repeated measures, to assess differences in He across locations. When overall differences were significant, post hoc pairwise Wilcoxon tests were conducted, and *p*-values were adjusted for multiple comparisons using the Benjamini–Hochberg false discovery rate (FDR) correction [83]. Statistical analysis and data visualization were performed in R v4.5.0 [86], using the packages tidyverse v1.3.0 [87] and rstatix v0.7.2 [88]. Unless otherwise indicated, results are presented as mean ± standard error (SE).

### 2.6. Kinship Inference and Relatedness Estimation Framework

We used ML-RELATE [89] to estimate maximum-likelihood relatedness coefficients (*r*) and assign relationship categories among dyads (i.e., pairwise comparisons) of Pampas cat individuals sampled in the MSPV. This program was selected for its accuracy in estimating relatedness, particularly in resolving common relationships such as parent-offspring and full siblings [90,91]. ML-RELATE assigns each dyad to one of four relationship categories (unrelated [U], half-siblings [HS], full-siblings [FS], or parent–offspring [PO]) based on likelihood ratio tests. Expected relatedness coefficients range from 0.00 for unrelated individuals to ≥0.50 for first-order relationships (i.e., PO and FS). Complementary estimates of relatedness using the unbiased Wang estimator for small samples in program COANCESTRY [92] were computed. Pairwise relationships assigned by ML-RELATE were visualized using a chord diagram in R v4.5.0 [86], employing the packages igraph v 2.1.4 [93] and ggraph v2.1.0 [94].

### 2.7. Population Size and Bottleneck Estimation Framework

Census population size (Nc) was estimated using the CAPWIRE approach [95], a capture–mark–recapture framework specifically developed for noninvasive genetic sampling of small populations. CAPWIRE assumes demographic closure and allows sampling with replacement, enabling multiple detections of individuals within a single sampling session. All surveys were treated as a single session. Analyses were conducted in the R package capwire v1.3. [96]. Two alternative models were evaluated: the Equal Capture Model (ECM), which assumes homogeneous detection probabilities, and the Two-Innate Rates Model (TIRM), which accounts for capture heterogeneity. Model selection was performed using a parametric bootstrap likelihood ratio test (LRT) with 100 replicates, with a significant LRT (*p* < 0.05) supporting selection of TIRM over ECM. Based on preliminary results, the maximum population size was constrained to 25 individuals. Final Nc estimates and 95% confidence intervals were obtained using 1000 parametric bootstrap replicates.

Effective population size (Ne) was estimated using the linkage disequilibrium (LD) method implemented in NeEstimator v2.0 [97]. This method infers Ne from nonrandom associations among alleles at different loci and assumes selectively neutral markers and random mating. All sampling seasons were pooled into a single session under the closed-population assumption. To reduce upward bias associated with rare alleles, a minimum allele frequency threshold of 0.02 was applied.

To detect signatures of recent genetic bottlenecks, we used BOTTLENECK v1.2.02 [98], which identifies deviations from mutation–drift equilibrium based on heterozygosity excess [16,99]. Analyses were conducted under three mutation models: the Infinite Allele Model (IAM), the Stepwise Mutation Model (SMM), and the Two-Phase Model (TPM), which incorporates features of both IAM and SMM to better reflect microsatellite evolution [100]. For TPM analyses, variance was calculated over 1000 iterations using a mutation composition of 70% SMM and 30% IAM. Statistical significance of heterozygosity excess was assessed using the Wilcoxon signed-rank test [16]. We additionally applied the mode-shift test to evaluate distortions in allele frequency distributions [15,100]; under mutation–drift equilibrium, an L-shaped distribution is expected, whereas a shifted mode indicates recent loss of low-frequency alleles consistent with population decline [14,16].

Finally, M-ratio values were calculated across all microsatellite loci in R v4.5.0 [86], following the method of [14]. This index measures the ratio of the number of alleles to the range in allele sizes, with lower values indicating a reduction in rare alleles associated with population bottlenecks [99,101]. The M-ratio test is particularly effective for detecting historical reductions in effective population size before allele frequencies reach equilibrium [15]. An M-ratio below the critical threshold (Mc = 0.68) is considered indicative of a recent bottleneck event [14].

### 2.8. Dietary Composition Metrics and Multivariate Analysis Framework

Diet composition for the Pampas cat was quantified using frequency of occurrence (FOO) and weighted percent of occurrence (wPOO). FOO represents the percentage of samples containing a specific prey item, while wPOO reflects its proportional contribution within each sample, averaged across all samples [37,102]. Although FOO is a conservative occurrence-based measure, it can overemphasize rare food items because both dominant and infrequent prey are weighted equally [35]. To address this limitation, wPOO weights each detection by the number of prey taxa in a sample, ensuring equal influence among samples and providing a more biologically realistic estimate of each item’s proportional contribution [102,103].

We examined individual diets to determine whether some cats showed preferences for particular prey types. In addition, we evaluated sex-based dietary composition using Bray–Curtis dissimilarities and permutational multivariate analysis of variance (PERMANOVA) with false discovery rate (FDR) correction [34]. Data processing and visualization were conducted in R v4.5.0 [86], and dissimilarity patterns were visualized via non-metric multidimensional scaling (NMDS) using the metaMDS function from the package vegan v2.6-4 [104].

## 3. Results

### 3.1. Genotyping Success and Individual Identification

Noninvasive genetic sampling and microsatellite genotyping enabled individual identification and recapture monitoring of Pampas cats across three consecutive sampling years in the MSPV. A total of 142 fecal samples were collected during three sampling periods: 2019 (*n* = 46), 2020 (*n* = 78), and 2021 (*n* = 18). Nuclear DNA amplification at 11 microsatellite loci yielded a genotyping success rate of 48%, resulting in 68 successfully genotyped samples (2019: *n* = 12; 2020: *n* = 52; 2021: *n* = 4). Cumulative genotyping error rates were low, averaging 0.005 for false alleles and 0.21 for allelic dropout, with per-locus rates ranging from 0.00 to 0.37. Loci exhibiting genotyping irregularities were further evaluated during quality control analyses, and three loci were subsequently removed prior to downstream population genetic analyses to ensure robustness of individual identification and parameter estimation. The probability of identity (P_(ID)_ = 4.0 × 10^−8^) and probability of identity siblings (P_(ID)sibs_ = 4.6 × 10^−4^) across all loci indicated high discriminatory power. Additionally, only six or seven loci were required to reach the P_(ID)_ (5.5 × 10^−4^) and P_(ID)sibs_ (8.3 × 10^−3^) thresholds (*p* < 0.01), respectively, confirming the panel’s effectiveness for individual identification.

GenAlEx analysis identified nine unique multilocus genotypes, comprising six males and three females. Based on the 3013-ha area of the MSPV, this corresponds to an estimated density of ~0.30 individuals/km^2^. The overall sex ratio was male-biased at 2:1, with minor interannual variation: three males and two females were detected in 2019, six males and three females in 2020, and two males and two females in 2021. Most individuals were recaptured in at least one subsequent sampling year, although capture histories exhibited apparent sex-based differences. Female detections were consistent, with F1 recorded across all three years (2019–2021), F2 in 2019–2020, and F3 in 2020–2021, reflecting multi-year persistence and high site fidelity. In contrast, male detections were more variable: M1–M3 were recaptured in 2020 but not detected in 2021, M4 was recorded only in 2020, and M5–M6 were first identified in 2020 and subsequently recaptured in 2021. The 2020 sampling season yielded the highest number of unique individuals (*n* = 9), coinciding with the largest sampling effort and representing the only year in which all genotypes were detected (Figure S1).

### 3.2. Genetic Diversity Patterns and Regional Comparisons

Quality control analyses identified three loci with genotyping irregularities that were removed prior to downstream analyses. Examination of the complete dataset (Table S2) revealed a high frequency of null alleles at FCA132 (FNA = 0.33), the only locus showing a significant deviation from HWE after Benjamini–Hochberg correction (*p* = 0.032). Additionally, loci FCA008 and FCA294 displayed unusually high observed heterozygosity (Ho = 1.00) and strongly negative inbreeding coefficients (F_IS_ = −0.42 and −0.31, respectively), suggesting potential scoring inconsistencies. Consequently, the final dataset comprised eight loci used for analyses of genetic diversity, relatedness, and recent bottlenecks. Based on the eight retained loci, the Pampas cat population from the MSPV exhibited moderate genetic diversity. The mean Na was 3.25 ± 0.37, and AR of 3.47 ± 0.32, while mean observed and expected heterozygosity values were Ho = 0.69 ± 0.07 and He = 0.58 ± 0.04, respectively. All loci were polymorphic, although FCA031 and FCA117 showed reduced allelic variation, each possessing only two alleles (Table 1).

**Figure 2:**
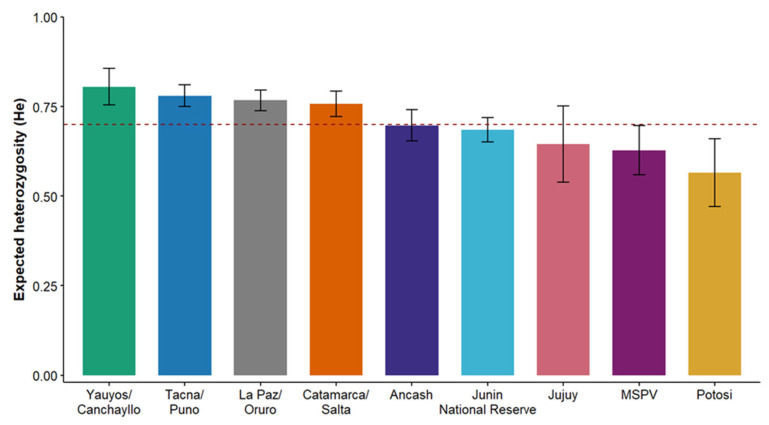
Comparison of expected heterozygosity (He ± SE) across Pampas cat (*L. garleppi*) populations from four regions: central Peru (Ancash, Yauyos/Canchayllo, Junin National Reserve, Tacna/Puno), northwestern Peru (MSPV), western Bolivia (La Paz/Oruro, Potosí), and northern Argentina (Jujuy, Catamarca/Salta). Estimates were derived from a per-locus dataset including the only four microsatellite loci shared across studies (FCA031, FCA045, FCA096, and FCA294; [85]). The red dashed line denotes the overall mean He (~0.70) across all localities.

Regional comparisons based on the four microsatellite loci shared across localities revealed moderate but nonsignificant variation in He among Pampas cat populations. Mean He ranged from 0.57 ± 0.09 in Potosí to 0.81 ± 0.05 in Yauyos/Canchayllo (Figure 2), with intermediate values in Tacna/Puno (0.78 ± 0.03), La Paz/Oruro (0.77 ± 0.03), and Catamarca/Salta (0.76 ± 0.04). Lower values were observed in Ancash (0.70 ± 0.04), Junín National Reserve (0.69 ± 0.03), MSPV (0.63 ± 0.07), and Jujuy (0.65 ± 0.11). Despite this range, the Friedman test indicated no statistically significant differences in He among localities (χ^2^_(8)_ = 13.3, *p* = 0.101), and all pairwise Wilcoxon comparisons remained nonsignificant after false-discovery-rate correction (Table S3; all *p* ≥ 0.529).

### 3.3. Kin Structure and Relatedness Patterns

**Figure 3:**
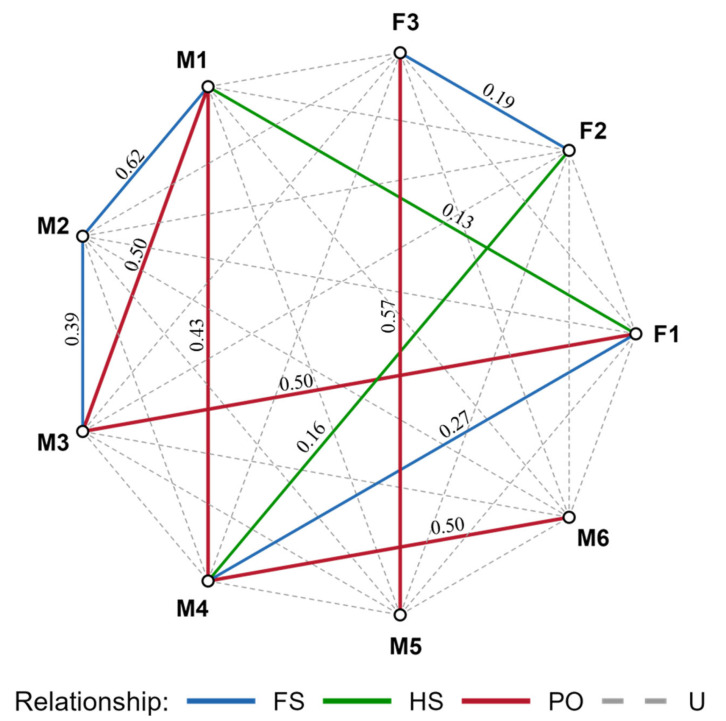
Chord diagram of pairwise relationships among nine Pampas cats (*L. garleppi*) individuals sampled in the MSPV, as inferred by ML-RELATE. Relationship categories are shown in the upper triangle: parent–offspring (PO, red), full siblings (FS, blue), half siblings (HS, green), and unrelated (U, dashed gray).

Pairwise analyses revealed strong kin associations within the MSPV Pampas cat population, with ML-RELATE and COANCESTRY yielding highly congruent results. Most individuals were connected through close kin dyads, dominated by FS and PO relationships, suggesting a multi-generational kin structure within the population (Figure 3). ML-RELATE identified four FS dyads (M2–M1, M3–M2, F1–M4, F3–F2) with relatedness coefficients (*r*) ranging from 0.19 to 0.62, and five PO dyads (M3–M1, M4–M1, F1–M3, M4–M6, F3–M5) exhibiting higher relatedness (*r* = 0.43–0.57). These findings were supported by likelihood comparisons, with clear rejection of alternative categories (ΔLnL ≥ 0.73; unsupported alternatives = 9999). Two dyads (F1–M1 and F2–M4) were classified as HS and showed comparatively lower coefficients (*r* = 0.13–0.16) and weaker support. Complementary estimates using the Wang estimator in COANCESTRY broadly supported the relationships inferred by ML-RELATE, although relatedness coefficients were generally lower and thus slightly more conservative (Table S4).

### 3.4. Population Size Estimates and Bottleneck Evidence

Estimates of census (Nc) and effective population size (Ne), together with multiple bottleneck detection approaches, indicate that the Pampas cat population at MSPV is small and has recently undergone a demographic contraction. The Two-Innate Rates Model (TIRM) implemented in CAPWIRE generated an Nc estimate of 9 individuals (95% CI: 7.0–9.0) and provided a significantly better fit than the Equal Capture Model (ECM; LRT, *p* = 0.0099; Table S5), although both models yielded similar point estimates. The effective population size was markedly low (Ne = 2.4; 95% CI: 1.5–7.4), consistent with reduced genetic variation. Bottleneck analyses further supported this pattern: the Wilcoxon signed-rank test detected significant heterozygosity excess under IAM (*p* = 0.00195), TPM (*p* = 0.00195), and SMM (*p* = 0.00977), with 7–8 loci exceeding mutation–drift expectations. The mode-shift test revealed a shifted allele-frequency distribution rather than the typical L-shaped profile expected under equilibrium (Table 2). Consistent with these results, the mean M-ratio across loci (0.37) was well below the critical threshold (Mc = 0.68), collectively providing robust evidence of a recent reduction in effective population size and loss of allelic diversity in the MSPV population.

### 3.5. Trophic Composition and Dietary Patterns

The dietary analysis of Pampas cats in the MSPV indicated a trophic profile predominantly composed of rodents, with no observed differences in prey composition based on sex. Out of the 53 fecal samples examined, 41 (77%) were successfully amplified and met the quality control criteria for dietary assessment. In addition, eight vertebrate prey species across six families, five orders, and two classes—Aves and Mammalia were identified (Table 3). Avian prey represented a diverse assemblage spanning four orders: Anseriformes, Gruiformes, Pelecaniformes, and Passeriformes. Among birds, the mangrove rail (*Rallus longirostris*) was most frequently detected (%FOO = 20%), contributing 12% to the weighted percent of occurrence (wPOO). The rufous-necked wood rail (*Aramides axillaris*, %FOO = 12%, wPOO = 8%) and common moorhen (*Gallinula chloropus*, %FOO = 10%, wPOO = 10%) were also frequently consumed. In contrast, the cinnamon teal (*Spatula cyanoptera*), roseate spoonbill (*Platalea ajaja*), and many-colored rush-tyrant (*Tachuris rubrigastra*) were less prevalent in the diet (≤10% FOO; ≤7% wPOO). Mammalian prey was dominated by the yellowish rice rat (*A. xanthaeolus*), detected in 59% of samples and accounting for 53% of the wPOO. The house mouse (*Mus musculus*) was detected occasionally (%FOO = 5%, wPOO = 2%). Although data were collected over three years, we did not perform year-specific dietary comparisons due to limited sample sizes in 2019 (*n* = 8) and 2021 (*n* = 3).

Dietary patterns varied across individuals, with *A. xanthaeolus* emerging as the dominant prey in both frequency of occurrence (Table S6) and weighted percent of occurrence (Table S7). For example, among females, F1 (*n* = 8) and F2 (*n* = 6) consumed *A. xanthaeolus* frequently (%FOO = 10–12%) and in high proportions (wPOO = 54–67%). F1 also incorporated *T. rubrigastra* (%FOO = 5%, wPOO = 25%) and *R. longirostris* (%FOO = 2%, wPOO = 13%), whereas F3 (*n* = 2) presented an even split between *A. xanthaeolus* and *R. longirostris* (wPOO = 50% each). However, the latter pattern should be viewed with caution due to the small sample size. Among males, M4 (*n* = 6) showed the strongest specialization, with *A. xanthaeolus* detected in all scats (%FOO = 15%) and 100% of wPOO. M5 (*n* = 7) also relied heavily on rodents (%FOO = 12%, wPOO = 43%) but incorporated waterbirds, including *R. longirostris* (%FOO = 10%, wPOO = 29%) and *A. axillaris* (%FOO = 2%, wPOO = 14%). M3 (*n* = 5) exhibiting the most diverse avian diet, with *S. cyanoptera* (%FOO = 5%, wPOO = 40%) and *P. ajaja* (%FOO = 2%, wPOO = 20%) alongside rodents. M2 (*n* = 2) consumed *A. xanthaeolus* (%FOO = 2%, wPOO = 50%) and *R. longirostris* (%FOO = 2%, wPOO = 25%), while *G. chloropus* (%FOO = 2%, wPOO = 100%) was the only item reported for M6 (*n* = 1).

**Figure 4:**
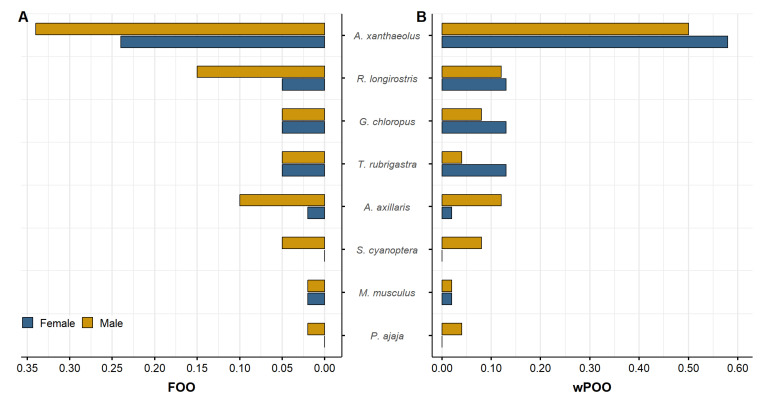
Sex-specific diet composition of Pampas cats (*L. garleppi*) in the MSPV, based on DNA metabarcoding of fecal samples. Panel (**A**) shows the Frequency of Occurrence (FOO), representing the proportion of scats containing each prey taxon. Panel (**B**) shows the Weighted Percent of Occurrence (wPOO), which accounts for multiple prey items per scat and highlights their relative dietary contribution. Bars represent females (blue) and males (golden).

**Figure 5:**
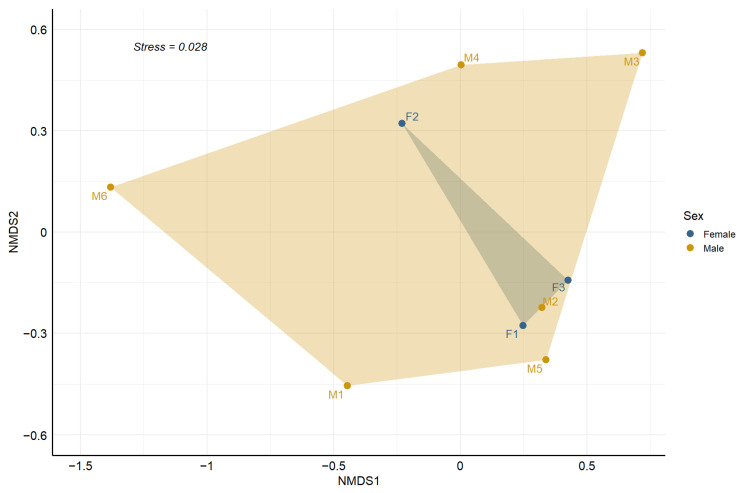
Non-metric multidimensional scaling (NMDS) ordination of Pampas cat (*L. garleppi*) diet composition based on Bray–Curtis dissimilarity, illustrating sex-specific variation in trophic niches within the MSPV. Individuals are grouped by sex: females (blue) and males (orange). Shaded polygons delineate convex hulls enclosing individuals of the same sex.

Sex-specific analyses revealed comparable dietary compositions, with *A. xanthaeolus* dominating prey profiles for both sexes (Figure 4). This rodent was detected in 24% of female samples (wPOO = 58%) and 34% of male samples (wPOO = 50%). Males exhibited broader prey diversity, with higher consumption of *R. longirostris* (%FOO = 15%, wPOO = 12%) and *A. axillaris* (%FOO = 10%, wPOO = 12%). Conversely, females showed more even consumption of *G. chloropus*, *R. longirostris*, and *T. rubrigastra*, each appearing in 5% of samples (wPOO = 13%). Prey items, such as *M. musculus* and *P. ajaja* were rare in the diet of both sexes, with slightly higher occurrence in male diets. The non-metric multidimensional scaling (NMDS) based on Bray–Curtis dissimilarities revealed partially overlapping dietary profiles between sexes (stress = 0.028). Convex hulls surrounding individuals of each sex indicated moderate dispersion among males and tighter clustering among females. In addition, visual inspection showed no clear separation between male and female diets. This qualitative pattern aligns with PERMANOVA results, which revealed no significant sex-based differences in diet composition (R^2^ = 0.066, F = 0.494, *p* = 0.761; Figure 5).

## 4. Discussion

### 4.1. Individual Identification and Sex-Specific Population Dynamics

Noninvasive genetic sampling at the MSPV enabled sex identification and multi-year tracking of nine *L. garleppi* individuals, providing one of the first longitudinal insights into population persistence for this species. Overall, the capture–recapture data support our expectation of sex-specific variation in apparent sex ratio and persistence driven by movement ecology, although some predictions were only partially met. Detections were male-biased (~2:1) across the three sampling years, consistent with sex-specific differences in space use and detectability. As predicted, females exhibited greater persistence over time: F1 was detected in all sampling periods, supporting high site fidelity and female-biased philopatry typical of small felids [47]. Although F2 and F3 showed shorter occupancy periods, their temporal overlap suggests localized retention within the system, even if territory tenure may be influenced by ecological or social factors [28,106]. In contrast, males displayed greater interannual variability in detections, consistent with our expectation of higher turnover: M1–M3 were not detected after 2020, M4 was recorded only during that year, and M5 and M6, first identified in 2020, persisted into 2021. This pattern aligns with male-biased dispersal, mortality, or temporary emigration [107], and supports the predicted sex-specific dynamics under restricted connectivity. Although we cannot exclude the possibility that some individuals were present but undetected, the overall pattern indicates greater temporal instability among males relative to females.

Male-biased dispersal typically reduces local competition and minimizes inbreeding within populations [108]. However, unsuccessful dispersal in human-altered landscapes or across natural barriers like the Sechura Desert may restrict movement and promote temporary returns to natal areas [47]. Although the ages of sampled Pampas cats were unknown, studies of other small felids indicate that dispersal generally occurs ~12 months of age [109,110], a pattern likely shared by this species. Yet reduced connectivity may delay or prevent dispersal, as observed in ocelots (*L. pardalis*) and pumas (*Puma concolor*), where landscape barriers restrict gene flow and can result in extended male residency within or near natal territories [28,111]. In our study, the absence of unrelated genotypes across three consecutive years, combined with repeated recaptures of close relatives, supports the hypothesis that the MSPV population is demographically closed with limited or no immigration. Under this expectation, we predicted relatively high interannual recapture rates, with most individuals identified in 2019–2020 expected to be detected again in 2021. However, this prediction was only partially supported. Interannual recapture rates were 20% and 44%, respectively, indicating lower persistence than anticipated, particularly among males. This discrepancy suggests that turnover, driven by mortality, temporary emigration, or undetected dispersal, may be higher than expected even within a system characterized by restricted immigration.

### 4.2. Genetic Diversity, Isolation, and Demographic History

Although the MSPV population of *L. garleppi* is small and apparently isolated, it retains moderate genetic diversity, suggesting historical connectivity and demographic stability. The finalized eight microsatellite loci panel revealed mean heterozygosity levels (Ho = 0.69 ± 0.07; He = 0.58 ± 0.04) higher than expected for an isolated population, where genetic drift typically reduces allelic diversity [4,112]. However, several factors may explain this pattern. For instance, the founding group might have carried high genetic variation and maintained sufficient effective size to preserve diversity through time [113].

Alternatively, moderate historical gene flow could have enriched genetic diversity before isolation, leaving a legacy that persists despite limited contemporary immigration [114]. Additionally, behavioral traits such as solitary habits, overlapping ranges, and possible multiple paternity might also boost heterozygosity by promoting local genetic mixing [115]. Finally, limited male movement within the system may shuffle alleles among neighboring territories, slowing drift-driven erosion [116]. It is also possible that occasional migrants were not detected during field sampling, particularly given unequal sampling effort across years and the low probability of encountering transient individuals. Nevertheless, without ongoing immigration, genetic diversity is unlikely to be replenished and will gradually decline through drift and inbreeding [114,117], emphasizing the need for continuous genomic monitoring.

Comparative analyses across the four microsatellite loci shared with earlier studies [85] revealed also moderate but spatially structured genetic diversity among Pampas cat populations, with most localities clustering around the overall mean (He = 0.70). Although the Friedman test detected no significant differences in He among sites, a subtle regional gradient was evident. For instance, the MSPV population exhibited one of the lowest mean He values (0.63 ± 0.07) compared with central Peruvian (mean He = 0.69–0.81) and northern Argentine populations (mean He = 0.65–0.76). This discrepancy likely reflects historical founder effects and limited connectivity with Andean source populations. These results support our hypothesis that MSPV operates as a demographically closed population with limited immigration, leading to reduced genetic diversity relative to other regions. The observed pattern is also consistent with the center–periphery hypothesis, which predicts that small, geographically peripheral populations experience reduced genetic variation due to limited dispersal and intensified drift, resulting in a progressive decline in diversity and increased inbreeding [112,118,119].

The spatial heterogeneity in He among localities highlights how geographic context and historical connectivity have jointly shaped the genetic structure of *L. garleppi*, underscoring the heightened susceptibility of peripheral populations to isolation and genetic erosion [120,121]. In addition, the persistence of moderate heterozygosity across populations likely reflects residual gene flow or male-mediated dispersal that has mitigated the loss of allelic diversity despite recent fragmentation [113,116,117]. However, the stability of this variation may be transient, as continued habitat degradation and demographic stochasticity can accelerate drift and inbreeding in small, peripheral populations [4,9,114]. Consequently, MSPV should be regarded as a genetically constrained but evolutionarily important population, where maintaining functional corridors and facilitating natural dispersal are essential to safeguard its long-term genetic resilience across Peru’s fragmented coastal landscapes.

### 4.3. Kin-Structured Population Dynamics and Inbreeding Risk

The high frequency of close-kin dyads observed among Pampas cats in the MSPV indicates a strongly interconnected kin network, consistent with limited dispersal and high spatial cohesion within this coastal population. All individuals had first-order relationships as detected by ML-RELATE and confirmed by COANCESTRY, including four FS dyads (M2–M1, F1–M4, M3–M2, and F3–F2) and five PO pairs (M3–M1, M4–M1, F1–M3, F3–M5, and M4–M6). The concordance between estimators increased confidence in kinship assignments, although the Wang estimator yielded slightly lower *r*-values, a pattern also reported in ocelot studies from Costa Rica [67]. Kin clustering was particularly evident among female-associated dyads (e.g., F1–M1, F1–M3, F1–M4, F2–M4, F3–M5, and F3–F2), supporting the presence of matrilineal site fidelity. Similar female-centered social structures occur in solitary felids such as Eurasian lynx (*Lynx lynx*) and ocelots, where spatial overlap is greater among related individuals [28,122]. Such kin-structured spatial organization, particularly in philopatric females, may enhance cooperation, tolerance, and access to resources but can also intensify genetic relatedness within small populations [123].

Although male Pampas cats are generally expected to disperse [124], the presence of several male kin dyads, including FS (M2–M1, M3–M2), and PO pairs (M3–M1, M4–M1, M4–M6) suggests that dispersal opportunities are limited. These restrictions may be reinforced by natural barriers or human-related pressures such as habitat conversion, road networks, and livestock activity [47,111,125]. The spatial clustering of related individuals in MSPV raises significant conservation concerns, particularly given the small population size, recent bottleneck signal, and absence of unrelated genotypes. Populations dominated by kin dyads are vulnerable to inbreeding, which can reduce reproductive success, increase the expression of deleterious alleles, and erode adaptive potential [47,126]. Additionally, the co-occurrence of multiple first-order relatives within a limited area implies a high probability of kin mating. Furthermore, ongoing habitat degradation from grazing, fire, and agricultural expansion [41,127] likely limits dispersal and gene flow, exacerbating these genetic risks and increasing the likelihood of demographic instability.

Conservation strategies should prioritize restoring and maintaining habitat connectivity to address the strong kin structure observed in MSPV. Protecting riparian corridors and lowland passages that connect coastal mangroves with inland or highland populations, where distinct alleles have been reported [85], would promote dispersal and reduce inbreeding. Additionally, long-term genetic monitoring is critical to detect changes in kin structure over time, track potential immigration events, and support adaptive management [128]. Evidence from Eurasian lynx and pine martens (*Martes martes*) shows that maintaining landscape permeability increases genetic exchange and improves population persistence in fragmented environments [129,130]. In this context, proactive landscape management, including riparian restoration and reducing human disturbance, is essential to preserve the evolutionary potential of *L. garleppi* in this coastal ecosystem. By addressing the combined effects of small population size, isolation, and kin clustering, conservation efforts can help sustain genetic diversity and reduce extinction risk in one of the species’ most peripheral and ecologically distinctive populations.

### 4.4. Demographic Contraction and Genetic Erosion

Integrated demographic and genetic analyses reveal that the MSPV Pampas cat population is critically small and undergoing ongoing genetic erosion consistent with a recent population bottleneck. Consistent with this pattern, CAPWIRE analyses estimated a census size (Nc) of nine individuals (95% CI: 7.0–9.0), with the TIRM significantly outperforming the ECM (LRT, *p* = 0.0099), indicating heterogeneous detection probabilities potentially linked to sex-specific movement or territorial behavior [95,96]. The effective population size was markedly low (Ne = 2.4; 95% CI: 1.5–7.4), yielding an Ne/Nc ratio of ~0.27, comparable to values reported for other small, structured felid populations undergoing rapid genetic drift, including ocelots in southern Texas (mean Ne/Nc = 0.29 [131]) and the North China leopard (*Panthera pardus japonensis*; Ne/Nc = 0.30 [132]). These results fully support our hypothesis that the MSPV population would be small in both census and effective size and exhibit signals of a recent bottleneck. Although genetic diversity remains moderate (Figure 2), this pattern likely reflects recent isolation or historical connectivity rather than demographic stability, as observed in bottlenecked populations such as the Eurasian wolf (*Canis lupus lupus*), Iberian lynx (*Lynx pardinus*), and Eurasian lynx, where diversity erosion often lags behind demographic contraction [133,134]. Nevertheless, the combination of low Nc and Ne suggests that even minor stochastic events, such as disease outbreaks or extreme climatic conditions could substantially compromise population viability [9,11].

Genetic bottleneck analyses further corroborate a recent and significant demographic contraction in the MSPV population. The Wilcoxon signed-rank test detected significant heterozygosity excess under all mutation models (IAM, TPM, and SMM), with 7–8 loci exceeding mutation–drift expectations [16,95,97]. The mode-shift test revealed a deviation from the typical L-shaped allele-frequency distribution expected under equilibrium, consistent with recent loss of low-frequency alleles [15]. Likewise, the mean M-ratio (0.37) fell well below the critical threshold (Mc = 0.68), indicating historical erosion of allelic richness and slow recovery following contraction [14]. Such genetic reductions are known to elevate extinction risk by decreasing adaptive potential and increasing mutational load [4,135,136]. Similar erosion patterns observed in the Iberian lynx and Florida panther (*P. c. coryi*) preceded successful genetic rescue through translocations and habitat reconnection [137,138], while studies on black bears (*Ursus americanus*) and dingoes highlight the potential for quick recovery if genetic exchange is restored [136,139]. Collectively, the convergence of low Nc, severely reduced Ne, and multiple independent bottleneck signals indicates that the MSPV Pampas cat population is experiencing ongoing genetic erosion and will require proactive management to restore connectivity and ensure long-term viability.

### 4.5. Trophic Ecology and Prey-Use Dynamics

Pampas cats in the MSPV primarily prey on rodents and wetland-associated birds, reflecting the high local abundance of small mammals and aquatic birds in this coastal mangrove mosaic. This trophic composition supports our hypothesis that MSPV individuals exhibit a foraging strategy adapted to locally abundant prey, particularly rodents and birds. Notably, the dominance of *A. xanthaeolus* in over half of all samples, along with the frequent occurrence of marsh-dependent species such as *R. longirostris* and *A. axillaris*, suggests a foraging strategy closely tied to habitats that promote stable rodent populations and diverse bird communities [140,141]. Similar rodent-dominated diets have been reported for *L. garleppi* in the high-Andean regions of Peru and Argentina [50,141], and comparable hunting patterns have been observed in other Leopardus species and small felids, such as the jaguarundi (*Puma yagouaroundi* [140,142,143]).

Several authors have emphasized the significant role that Cricetidae rodents play for small felids, providing most of their daily energy requirements and contributing to reproductive success [144,145,146]. In contrast, birds are generally considered secondary or opportunistic prey, contributing less consistently to energy budgets [147,148]. In this context, our findings underscore the importance of conserving rodent-rich habitats, as they provide a consistent prey base and support trophic diversity to buffer seasonal fluctuations [50,142]. However, the occasional presence of *M. musculus* raises ecological concerns, as invasive rodents can disrupt trophic interactions, outcompete native prey, and increase disease transmission risks for carnivores [81,149]. Moreover, habitat loss and hydrological alterations can reduce prey abundance and diversity, triggering cascading effects that compromise predator health and threaten population stability within this vulnerable coastal ecosystem [150,151].

Dietary profiles suggested inter-individual variation in prey use within the MSPV population. However, the small and uneven number of scat samples per individual precludes formal inference about individual specialization, and the observed patterns should therefore be interpreted cautiously. Within these constraints, *A. xanthaeolus* was consistently dominant in both males and females, indicating a shared core prey resource within the population. Nevertheless, some individuals of both sexes were repeatedly associated with rodent-heavy detections, whereas others exhibited a broader prey spectrum that included wetland-associated birds (e.g., *R. longirostris*, *S. cyanoptera*, *P. ajaja*), reflecting variability in prey composition rather than discrete trophic strategies. Consequently, these results are best viewed as descriptive, representing inter-individual dietary variation under limited and uneven per-individual sampling, rather than evidence of true specialization or structured niche partitioning.

Consistent with the PERMANOVA results, we found limited evidence for sex-based dietary divergence in the MSPV population, indicating broadly overlapping prey use between males and females. Although males exhibited slightly greater dispersion in NMDS space and consumed a wider variety of bird species (e.g., *S. cyanoptera*, *P. ajaja*), these trends were not statistically significant, suggesting that both sexes exploit similar prey resources when availability is high. Additionally, the consistent dominance of *A. xanthaeolus* in the diets of both males and females underscores the critical role of this native rodent as a keystone prey species, paralleling rodent-dominated trophic patterns reported for Andean mountain cats (*L. jacobita*) and Pampas cats in high-altitude systems, where diets are similarly structured around viscachas (Chinchillidae) and other dominant small mammals [140,141]. These results indicate strong trophic dependence on a limited set of core prey resources rather than sex-structured niche differentiation. Therefore, long-term conservation efforts in MSPV should prioritize the protection of wetland–terrestrial ecotones, the management of invasive rodent species, and the mitigation of anthropogenic pressures that threaten key prey populations and habitat integrity.

### 4.6. Conservation Implications

Effective conservation of the MSPV Pampas cat requires integrated strategies that address its extreme demographic limitation and ongoing genetic erosion. The critically low census size (Nc = 9) and severely reduced effective population size (Ne = 2.4) indicate that the population operates near the threshold where genetic drift, inbreeding, and demographic stochasticity can rapidly erode evolutionary potential. The low Ne/Nc ratio (~0.27), recent bottleneck signatures, and pronounced kin structure, where all genotyped individuals were connected through first-order relationships, collectively elevate extinction risk by limiting the availability of unrelated mates and accelerating the loss of rare alleles essential for adaptive resilience [2,4]. Although moderate heterozygosity persists, it likely reflects demographic inertia rather than stability, and without restored gene flow, continued allelic erosion is expected. In this sense, safeguarding and restoring landscape connectivity through riparian and desert-scrub corridors is therefore crucial to facilitate dispersal, increase effective size, and buffer the population against drift and inbreeding. Similar connectivity-driven recovery has enhanced persistence in other small carnivores inhabiting fragmented systems [129,152,153]. Additionally, management should focus on mitigating anthropogenic disturbances, reducing fire frequency, and preventing overgrazing to maintain habitat permeability and support recolonization from neighboring populations.

Maintaining high-quality habitat within the mangrove–terrestrial ecotone is equally important to sustain demographic stability and prey availability. The strong dietary dependence on the native rodent *A. xanthaeolus* and marsh-associated birds (e.g., *R. longirostris*, *A. axillaris*) underscores the need to protect wetland–dryland interfaces and rodent-rich microhabitats that anchor the trophic web. Habitat degradation from livestock activity, fire, or hydrological alteration could reduce prey abundance and intensify demographic instability. Effective management should therefore prioritize vegetation restoration, hydrological balance, and the control of invasive species such as *M. musculus*, which can disrupt trophic interactions and introduce disease [149]. In addition, active community participation in mangrove conservation and sustainable land use can reduce human–wildlife conflict and strengthen compliance with Ramsar commitments, while simultaneously protecting critical foraging habitats and prey communities for this Pampas cat population.

Long-term conservation success for the Pampas cat population in the MSPV will rely on adaptive management guided by ongoing, science-based monitoring and evaluation. Annual or periodic noninvasive genetic surveys should evaluate kin structure, allelic diversity, and potential immigration, while diet metabarcoding can reveal seasonal shifts in prey composition and ecosystem health [25]. However, if restoring connectivity is inadequate, a carefully planned genetic rescue using ecologically compatible, pathogen-screened donor animals could be implemented accompanied by strict post-release monitoring. Success metrics, such as the recruitment of unrelated individuals, decreased bottleneck signals, and stable prey populations, should be established beforehand and integrated into local co-management plans. Additionally, complementary education and stewardship efforts would boost public engagement in habitat preservation and mangrove restoration. Together, these coordinated strategies will combine habitat restoration, demographic support, and genetic monitoring to protect *L. garleppi* at the edge of its range, ensuring both evolutionary survival and the ecological health of Peru’s northern coastal wetlands.

## Figures and Tables

**Table 1:** Summary statistics for 8 microsatellite loci genotyped and retained in nine Pampas cats (*L. garleppi*) from the MSPV, Peru, after removing loci FCA008, FCA132, and FCA294. Reported metrics include the total number of individuals detected (*n*), number of successfully genotyped and retained for analyses (N), probability of identity among siblings (P_(ID)sibs_), number of alleles (Na), allelic richness (AR), observed (Ho) and expected (He) heterozygosity, frequency of null alleles (FNA), genotyping error rate (GE), Weir and Cockerham’s inbreeding coefficient (F_IS_), tests of linkage disequilibrium (LD), standard error (SE), and *p*-values (*p*) from Hardy–Weinberg equilibrium (HWE) tests. Significant deviations from HWE after Benjamini–Hochberg correction (α = 0.05) are shown in bold.

Locus	Mangroves San Pedro de Vice (*n* = 9)										
	P_(ID)sibs_	N	Na	AR	Ho	He	FNA	GE	F_IS_	LD	*p*
F124	0.52	8	3	3.00	0.63	0.57	0.03	0.14	0.11	0.776	0.914
FCA031	0.63	8	2	2.00	0.38	0.43	0.00	0.15	0.06	0.953	1.000
FCA045	0.50	8	3	3.94	0.63	0.53	−0.04	0.16	−0.03	0.832	0.966
FCA075	0.46	8	4	3.94	0.75	0.67	−0.07	0.25	−0.10	0.796	0.966
FCA096	0.42	8	5	4.94	0.88	0.75	−0.03	0.37	−0.01	0.920	0.914
FCA117	0.55	8	2	2.94	0.50	0.50	−0.01	0.07	0.05	0.889	1.000
FCA126	0.49	8	3	3.00	0.88	0.57	−0.11	0.16	−0.19	0.774	0.914
FCA391	0.50	7	4	4.00	0.86	0.64	−0.11	0.27	−0.18	1.000	1.000
Overall	0.51	7.88	3.25	3.47	0.69	0.58	−0.04	0.20	−0.04	-	-
SE	0.02	0.13	0.37	0.32	0.07	0.04	0.02	0.03	0.04	-	-

**Table 2:** BOTTLENECK analysis for Pampas cats (*L. garleppi*) sampled in the MSPV, Peru. The table reports the observed and expected number of loci exhibiting heterozygosity excess under mutation–drift equilibrium. Results are provided for three mutation models: Infinite Allele Model (IAM), Two-Phase Mutation Model (TPM), and Stepwise Mutation Model (SMM). Statistical significance was assessed using the Wilcoxon signed-rank test (*p* = 0.05).

Model	Heterozygosity Excess			Allele Frequency Distribution
	Expected	Observed	*p*-Value	
IAM	4.46	8	0.00195	Shifted mode
TPM	4.51	8	0.00195	
SMM	4.72	7	0.00977	

**Table 3:** Avian and mammalian prey species detected in the diet of Pampas cats (*L. garleppi*) based on DNA metabarcoding of fecal samples collected between 2019 and 2021 in the MSPV, Peru. The table reports the frequency of occurrence (FOO) and weighted percent of occurrence (wPOO) for each prey taxon. N denotes the total number of fecal samples analyzed after quality filtering, while *n* indicates the number of samples in which each prey item was detected.

Class	Order	Family	Scientific Name	Common Name	*n*	FOO	wPOO
						(N = 41)	(N = 41)
Aves	Anseriformes	Anatidae	*S. cyanoptera*	Cinnamon teal	2	0.05	0.05
	Gruiformes	Rallidae	*A. axillaris*	Rufous-necked wood rail	5	0.12	0.08
			*G. chloropus*	Common moorhen	4	0.10	0.10
			*R. longirostris*	Mangrove rail	8	0.20	0.12
	Pelecaniformes	Threskiornithidae	*P. ajaja*	Roseate spoonbill	1	0.02	0.02
	Passeriformes	Tachurididae	*T. rubrigastra*	Many-colored rush-tyrant	4	0.10	0.07
Mammalia	Rodentia	Cricetidae	*A. xanthaeolus*	Yellowish rice rat	24	0.59	0.53
		Muridae	*M. musculus*	House mouse	2	0.05	0.02

Although the reference database matched sequences to *A. cajaneus*, this species is primarily distributed in central Peru [105] and has not been recorded in the MSPV. We therefore reassigned the sequences to *A. axillaris*, a congener documented at the site [57], to ensure taxonomic accuracy in local dietary reporting.

## Data Availability

The original contributions presented in this study are included in the article/Supplementary Materials. Further inquiries can be directed to the corresponding author.

## Supplementary Materials

The following supporting information can be downloaded at https://www.mdpi.com/article/10.3390/genes17030320/s1: Figure S1: Capture–recapture histories of nine Pampas cats (*L. garleppi*) monitored between 2019 and 2021 in the MSPV; Table S1: Nuclear microsatellite loci used for PCR amplification of fecal DNA samples from Pampas cats (*L. garleppi*) in the MSVP; Table S2: Summary statistics for 11 microsatellite loci genotyped in nine Pampas cats (*L. garleppi*) from the MSPV; Table S3: Pairwise Wilcoxon tests of expected heterozygosity (He) among Pampas cat localities using four shared microsatellite loci; Table S4: Maximum likelihood relatedness estimates among Pampas cats (*L. garleppi*) sampled in the MSPV; Table S5: Census population size (Nc) estimates for Pampas cats (*L. garleppi*) sampled in the MSPV; Table S6: Frequency of occurrence (FOO) of prey taxa detected in the diet of individual Pampas cats (*L. garleppi*) from the MSPV; Table S7: Weighted percent of occurrence (wPOO) of prey taxa detected in the diet of in-dividual Pampas cats (*L. garleppi*) from the MSPV.

## Abbreviations

The following abbreviations are used in this manuscript:

MSPV	Mangroves San Pedro de Vice
LEECG	Laboratory for Ecological, Evolutionary, and Conservation Genetics
FDR	Benjamini–Hochberg false discovery rate
IAM	Infinite Allele Model
SMM	Stepwise Mutation Model
TPM	Two-Phase Model
FOO	Frequency of occurrence
wPOO	Weighted percent of occurrence
