# Supplementary material for: Genetic Diversity, Demographic Parameters, and Trophic Ecology of the Pampas Cat (Leopardus garleppi) in a Ramsar Wetland of Northwestern Peru
Source: Genes (Basel). 2026 Mar 16;17(3):320. doi: 10.3390/genes17030320 (PMC13026185; doi:10.3390/genes17030320)
Supplement: Supplementary file 1 [file genes-17-00320-s001.zip › genes-4169655-supplementary.pdf]

Genetic diversity, demographic parameters, and trophic ecology of the pampas cat (*Leopardus garleppi*) in a Ramsar wetland of northwestern Peru

Materials and Methods

Microsatellite amplification and genotyping

We amplified microsatellite loci in a 7 µL reaction (total volume) containing 0.06 µL of double-deionized H_2O, 3.5 µL Qiagen Master Mix (Qiagen Inc.), 0.7 µL concentration Qiagen Q-solution (Qiagen Inc.), and 0.74 µL of primers (0.04 µL for F124 F and R, 0.05 µL for FCA008 F and R, 0.05 µL for FCA031 F and R, 0.05 µL for FCA045 F and R, 0.03 µL for FCA075 F and R, 0.13 µL for FCA096 F and R, 0.05 µL for FCA117 F and R, 0.07 µL for FCA126 F and R, 0.07 µL for FCA132 F and R, 0.05 µL for FCA294 F and R, 0.15 µL for FCA391 F and R), with all primers at 10µM concentration and 2.0 µL DNA extract.

Microsatellite PCR amplifications were performed on a Bio-Rad S1000™ Thermal Cycler (Bio-Rad Laboratories, Inc.) using the following thermal profile: an initial denaturation at 94 °C for 15 min; followed by a touchdown phase of 13 cycles consisting of 30 s at 94 °C (denaturation), 90 s at 62 °C with a 0.4 °C decrement per cycle (annealing), and 1 min at 72 °C (extension); then 32 cycles of 30 s at 94 °C, 90 s at 57 °C, and 1 min at 72 °C; concluding with a final extension of 30 min at 60 °C and a 10 min cooldown at 4 °C.

Table S1 Eleven nuclear microsatellite loci used for PCR amplification of fecal DNA from pampas cats (*L. garleppi*) in the MSPV, including fluorescent dye labels and final primer concentrations (μM) for each locus.

Locus	Dye	Concentration	Reference
F124	PET	0.04 μM	[1]
FCA008	FAM	0.06 μM	[2]
FCA031	VIC	0.06 μM	[2]
FCA045	VIC	0.06 μM	[2]
FCA075	VIC	0.03 μM	[2]
FCA096	FAM	0.14 μM	[2]
FCA117	FAM	0.06 μM	[2]
FCA126	PET	0.08 μM	[2]
FCA132	NED	0.08 μM	[2]
FCA294	PET	0.06 μM	[2]
FCA391	VIC	0.17 μM	[2]

Table S2 Summary statistics for 11 microsatellite loci genotyped in nine pampas cats (*L. garleppi*) from the MSPV, Peru. Reported metrics include the total number of individuals detected (n), number successfully genotyped and retained for analyses (N), probability of identity among siblings ($P_{(ID)sibs}$), number of alleles (Na), allelic richness (AR), observed (Ho) and expected (He) heterozygosity, frequency of null alleles (FNA), genotyping error rate (GE), Weir and Cockerham's inbreeding coefficient (F_{IS}), tests of linkage disequilibrium (LD), standard error (SE), and p-values (*p*) from Hardy–Weinberg equilibrium (HWE) tests. Significant deviations from HWE after Benjamini–Hochberg correction (α = 0.05) are shown in bold.

Locus	Mangroves San Pedro de Vice (n = 9)										
	$P_{(ID)sibs}$	N	Na	AR	Ho	He	FNA	GE	F_{IS}	LD	*p*
F124	0.52	9	3	2.99	0.56	0.59	0.02	0.14	0.11	0.761	0.739
FCA008	0.45	8	4	4.00	1.00	0.68	-0.19	0.31	-0.42	0.889	0.828
FCA031	0.63	9	2	2.00	0.44	0.44	0.00	0.15	0.06	0.946	1.000
FCA045	0.50	9	4	3.89	0.67	0.61	-0.03	0.16	-0.03	0.858	0.828
FCA075	0.46	9	4	3.89	0.78	0.67	-0.06	0.25	-0.10	0.812	0.830
FCA096	0.42	9	5	4.88	0.78	0.73	-0.03	0.37	-0.01	0.914	0.739
FCA117	0.55	9	3	2.89	0.56	0.55	0.00	0.07	0.05	0.934	1.000
FCA126	0.49	9	3	3.00	0.78	0.62	-0.10	0.16	-0.19	0.823	0.572
FCA132	0.60	9	2	2.00	0.00	0.49	0.33	0.00	1.00	0.836	**0.032**
FCA294	0.41	9	5	4.88	1.00	0.73	-0.15	0.36	-0.31	0.859	0.572
FCA391	0.50	8	4	4.00	0.75	0.60	-0.09	0.27	-0.18	0.960	1.000
Overall	0.50	8.82	3.55	3.49	0.66	0.61	-0.03	0.20	0.00	-	-

SE	0.02	0.11	0.29	0.28	0.08	0.03	0.04	0.04	0.10	-	-

Table S3 Post hoc pairwise Wilcoxon tests comparing expected heterozygosity (He) among Pampas cat sampling localities based on four shared microsatellite loci (FCA031, FCA045, FCA096, FCA294). *p*-values were adjusted using the Benjamini–Hochberg FDR correction (ns = not significant). Only sites with sample sizes ≥ 9 individuals were included.

Group 1	Group 2	*p*	*p*.adj	*p*_label
Yauyos/Canchayllo	Tacna/Puno	0.875	0.875	ns
Yauyos/Canchayllo	La Paz/Oruro	0.625	0.804	ns
Yauyos/Canchayllo	Catamarca/Salta	0.250	0.529	ns
Yauyos/Canchayllo	Ancash	0.250	0.529	ns
Yauyos/Canchayllo	Junin National Reserve	0.250	0.529	ns
Yauyos/Canchayllo	Jujuy	0.125	0.529	ns
Yauyos/Canchayllo	MSPV	0.125	0.529	ns
Yauyos/Canchayllo	Potosi	0.250	0.529	ns
Tacna/Puno	La Paz/Oruro	0.174	0.529	ns
Tacna/Puno	Catamarca/Salta	0.461	0.804	ns
Tacna/Puno	Ancash	0.181	0.529	ns
Tacna/Puno	Junin National Reserve	0.174	0.529	ns
Tacna/Puno	Jujuy	0.625	0.804	ns
Tacna/Puno	MSPV	0.125	0.529	ns
Tacna/Puno	Potosi	0.125	0.529	ns
La Paz/Oruro	Catamarca/Salta	0.875	0.875	ns
La Paz/Oruro	Ancash	0.250	0.529	ns
La Paz/Oruro	Junin National Reserve	0.250	0.529	ns
La Paz/Oruro	Jujuy	0.625	0.804	ns
La Paz/Oruro	MSPV	0.125	0.529	ns
La Paz/Oruro	Potosi	0.125	0.529	ns
Catamarca/Salta	Ancash	0.625	0.804	ns
Catamarca/Salta	Junin National Reserve	0.375	0.711	ns
Catamarca/Salta	Jujuy	0.789	0.875	ns
Catamarca/Salta	MSPV	0.125	0.529	ns
Catamarca/Salta	Potosi	0.250	0.529	ns
Ancash	Junin National Reserve	0.875	0.875	ns
Ancash	Jujuy	0.625	0.804	ns
Ancash	MSPV	0.625	0.804	ns
Ancash	Potosi	0.625	0.804	ns
Junin National Reserve	Jujuy	0.789	0.875	ns
Junin National Reserve	MSPV	0.875	0.875	ns
Junin National Reserve	Potosi	0.375	0.711	ns
Jujuy	MSPV	0.875	0.875	ns
Jujuy	Potosi	0.625	0.804	ns

MSPV	Potosi	0.875	0.875	ns

Table S4 Maximum likelihood relatedness estimates among Pampas cats (*L. garleppi*) sampled in the MSPV, based on 9 nuclear microsatellite loci. Pairwise relatedness coefficients (*r*) were calculated using ML-RELATE and COANCESTRY (Wang estimator). The sex of each individual is indicated as M (male) or F (female). Relationship categories assigned by ML-RELATE include unrelated (U), half-siblings (HS), full-siblings (FS), and parent–offspring (PO).

**Ind 1**	**Ind 2**	**Relatedness**		**Delta *Ln*(*L*)[a]**					
		**ML-RELATE**	**COANCESTRY**	**R[b]**	***LnL*(R)[c]**	**ΔU**	**ΔHS**	**ΔFS**	**ΔPO**
M2	M1	0.67	0.77	FS	-19.80	3.65	2.03	-	0.99
M3	M1	0.48	0.33	PO	-25.78	0.86	0.20	0.74	-
M3	M2	0.29	0.14	HS	-24.48	0.30	-	0.06	9999
M4	M1	0.50	0.21	PO	-26.04	0.73	0.24	1.21	-
F1	M1	0.37	0.27	HS	-23.73	0.23	-	0.52	0.01
F1	M3	0.50	0.54	PO	-23.82	0.78	0.28	0.82	-
F1	M4	0.37	0.46	FS	-23.82	0.90	0.63	-	9999
F3	F2	0.16	0.31	HS	-32.95	0.01	-	0.55	-
F3	M5	0.64	0.59	PO	-29.77	4.60	1.91	0.20	-
M6	M4	0.20	0.17	HS	-34.79	0.34	-	2.22	9999

[a] Delta *Ln*(*L*): The difference in log-likelihood values relative to the most likely relationship category. A Delta *Ln*(*L*) value of *9999* denotes that the corresponding relationship is not supported or is statistically implausible.

[b] *R*: The relationship category with the highest likelihood assignment in ML-RELATE.

[c] *LnL*(*R*): The log-likelihood associated with the most likely relationship (*R*) as inferred by ML-RELATE.

Table S5 Census population size (*N*c) estimates for pampas cats (*L. garleppi*) sampled in the MSPV, based on Capwire analysis. Estimates are presented under the Equal Capture Model (ECM) and the Two-Innate Rates Model (TIRM), with corresponding 95% confidence intervals (CI). Model fit was assessed using a likelihood ratio test (LRT), with statistical significance evaluated at $p < 0.05$ to determine the best-supported model.

**Two-Innate Rates Model (TIRM)**		**Equal Capture Model (ECM)**		**P-value**
Population size	95% CI	Population size	95% CI	
9	7.0 – 9.0	9	9.0 – 9.0	*0.0099*

Table S6 Frequency of occurrence (FOO) of prey taxa detected in the diet of individual pampas cats (*L. garleppi*) from the MSPV, based on DNA metabarcoding of fecal samples collected between 2019 and 2021. Values represent the proportional contribution of each prey species within scats, averaged across all samples for each individual. Sample size (n) denotes the number of scats analyzed per individual.

**Prey items**	**Female 1 (n = 8)**	**Female 2 (n = 6)**	**Female 3 (n = 2)**	**Male 1 (n = 4)**	**Male 2 (n = 2)**	**Male 3 (n = 5)**	**Male 4 (n = 6)**	**Male 5 (n = 7)**	**Male 6 (n = 1)**
Aegialomys xanthaeolus	0.12	0.10	0.02	0.02	0.02	0.05	0.15	0.10	
Spatula cyanoptera						0.05			
Aramides axillaris	0.02			0.05	0.02			0.02	
Gallinula chloropus		0.05		0.02					0.02
Mus musculus	0.02							0.02	
Platalea ajaja						0.02			
Rallus longirostris	0.02		0.02	0.02	0.02			0.10	
Tachuris rubrigastra	0.05							0.05	

Table S7 Weighted percent of occurrence (wPOO) of prey taxa detected in the diet of individual pampas cats (*L. garleppi*) from the MSPV, based on DNA metabarcoding of fecal samples collected between 2019 and 2021. Values represent the proportional contribution of each prey species within scats, averaged across all samples for each individual. Sample size (n) denotes the number of scats analyzed per individual.

**Prey items**	**Female 1 (n = 8)**	**Female 2 (n = 6)**	**Female 3 (n = 2)**	**Male 1 (n = 4)**	**Male 2 (n = 2)**	**Male 3 (n = 5)**	**Male 4 (n = 6)**	**Male 5 (n = 7)**	**Male 6 (n = 1)**
Aegialomys xanthaeolus	0.54	0.67	0.50	0.25	0.50	0.40	1.00	0.36	
Spatula cyanoptera						0.40			
Aramides axillaris	0.04			0.38	0.25			0.14	
Gallinula chloropus		0.33		0.25					1.00
Mus musculus	0.04							0.07	
Platalea ajaja						0.20			
Rallus longirostris	0.13		0.50	0.13	0.25			0.29	
Tachuris rubrigastra	0.25							0.14	

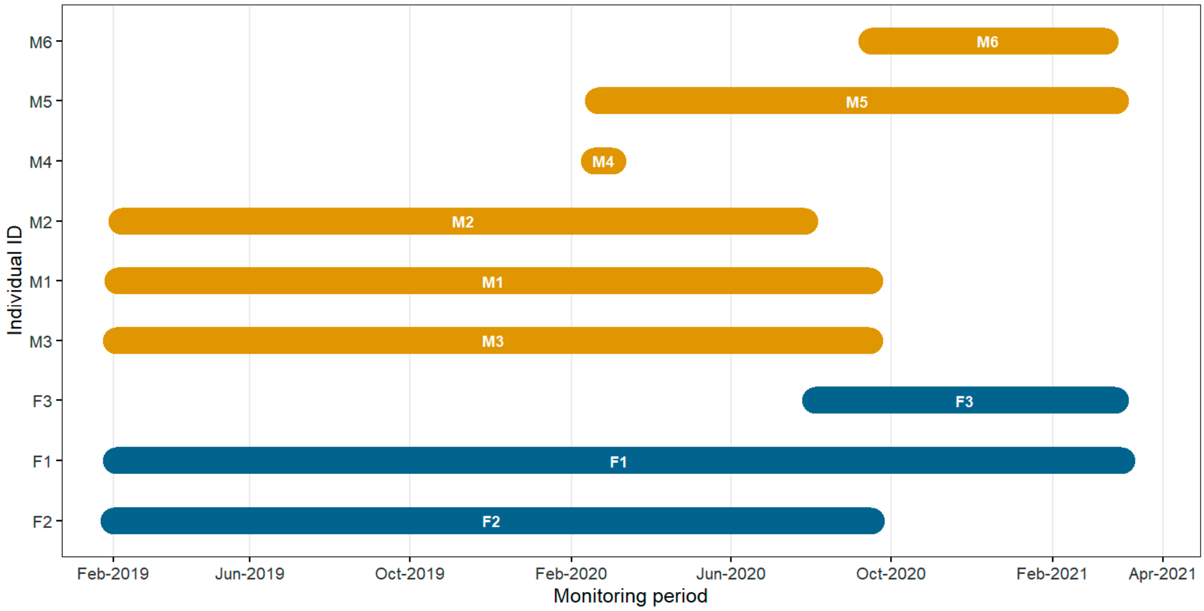

Figure S1 Capture–recapture histories of nine pampas cats (*L. garleppi*) monitored between 2019 and 2021 in the MSPV. Each horizontal bar represents the period between the first and last detection of an individual. Females (F1–F3) are shown in blue and males (M1–M6) in orange.

References

1. Menotti-Raymond, M.A.; David, V.A.; Wachter, L.L.; Butler, J.M.; O'Brien, S.J. An STR forensic typing system for genetic individualization of domestic cat (*Felis catus*) samples. *J. Forensic Sci.* **2005**, *50*, JFS2004317. https://doi.org/10.1520/JFS2004317
2. Menotti-Raymond, M.; David, V.A.; Lyons, L.A.; Schäffer, A.A.; Tomlin, J.F.; Hutton, M.K.; O'Brien, S.J. A genetic linkage map of microsatellites in the domestic cat (*Felis catus*). *Genomics* **1999**, *57*, 9–23. https://doi.org/10.1006/geno.1999.5743
